# Effect of physical activity on attention in school-age children with ADHD: a systematic review and meta-analysis of randomized controlled trials

**DOI:** 10.3389/fphys.2023.1189443

**Published:** 2023-07-27

**Authors:** Dong Li, Lan Li, Wanli Zang, Deng Wang, Chuyuan Miao, Chenmu Li, Li Zhou, Jin Yan

**Affiliations:** ^1^ School of Physical Education, Guangzhou Sport University, Guangzhou, China; ^2^ Department of International Culture Education, Chodang University, Muan, Republic of Korea; ^3^ Universuty of Maine at Presque Isle, Presque Isle, ME, United States; ^4^ Postgraduate School, University of Harbin Sport, Harbin, China; ^5^ LFE Research Group, Department of Health and Human Performance, Faculty of Physical Activity and Sport Science (INEF), Universidad Politécnica de Madrid (UPM), Madrid, Spain; ^6^ School of Nursing, Guangzhou Medical University, Guangzhou, China; ^7^ School of Sports and Health, Guizhou Medical University, Guiyang, China; ^8^ Centre for Active Living and Learning, University of Newcastle, Callaghan, NSW, Australia

**Keywords:** physical activity, children, ADHD, neurodevelopmental disorders, meta-analysis

## Abstract

**Background:** Attention problems are one of the core symptoms of Attention-deficit/hyperactivity disorder (ADHD) in children. Previous studies have shown that physical activity intervention has a positive impact on executive function in children and adolescents with ADHD, but there is limited research on attention problems in school-aged children with ADHD. There are still uncertainties about the appropriate physical activity interventions to improve attention problems in this population. This study conducted a Meta-analysis of randomized controlled trials (RCTs) related to physical activity intervention for attention problems in school-aged children with ADHD, providing a certain reference for precise intervention in attention problems for this population.

**Methods:** We systematically searched the following databases up to October 2022: PubMed, Embase, Web of Science, and Cochrane Library, to identify RCTs that investigated the effects of physical activity interventions on children with ADHD. Two investigators independently conducted literature screening, extraction, and quality assessment. We performed a meta-analysis using Stata 15.1.

**Results:** In total, we included 10 studies in this meta-analysis. The results indicated that physical activity intervention had a moderate effect in improving attention problems in school-aged children with ADHD (SMD = −0.48, 95% CI: 0.85, −0.07, *p* < 0.05). Furthermore, subgroup analysis showed that the effect of physical activity intervention was moderated by intervention type, frequency, and period, rather than the physical activity environment or single intervention time.

**Conclusion:** Our study suggests that cognitively engaging exercise is more effective in improving attention problems in school-aged children with ADHD. Specifically, when cognitive-engaging exercise is used as the type of physical activity and the intervention frequency is less than 3 times per week, with an intervention period of less than weeks, it is most beneficial for improving attention problems in school-aged children with ADHD. However, we should also consider individual differences in children with respect to their ADHD symptoms and accurately evaluate each child’s specific symptoms before intervention.

**Systematic Review Registration:** identifier (CRD42022363255).

## 1 Introduction

Attention deficit/hyperactivity disorder (ADHD) is a prevalent neurodevelopmental disorder that is characterized by challenges in attention, hyperactivity, or impulsive behaviour. It is usually diagnosed in school-aged children (Guha, 2014). According to the severity of symptoms of inattention, hyperactivity, or both, DSM-5 has identified three subtypes: inattentive subtype, hyperactive-impulsive subtype, and combined hyperactive-impulsive and inattentive subtype (Salvi et al., 2019). ADHD is more commonly diagnosed in boys than girls, almost three times as common (Faraone et al., 2021), and is often accompanied by some common comorbidities, such as autism spectrum disorder and oppositional defiant disorder (Tsujii et al., 2021). In addition, children with ADHD may experience related problems such as distractibility, impulse control, academic difficulties, and lack of physical activity (Benzing and Schmidt, 2019; Drigas and Karyotaki, 2019; Taylor et al., 2023).

Although ADHD is often diagnosed in school-age children, its behavioural problems can persist into adolescence and adulthood, thus impacting one’s entire life (Di Lorenzo et al., 2021). Moreover, over the long term, patients with ADHD symptoms incur significant social and economic costs. There is a correlation between ADHD symptoms and risks such as educational failure, difficulty managing interpersonal relationships, and criminal behaviour (Schein et al., 2022). The proportion of ADHD patients with poor academic performance, unemployment, and divorce rates are higher than that of the general population (Quenneville et al., 2022). In this regard, attention refers to the ability to sustain focus on information for a few seconds and is closely associated with working memory (Diamond, 2013). Previous research has demonstrated that attention significantly influences various domains such as language, literacy, and mathematics, making it a crucial determinant of academic achievement. The prevalent occurrence of attention issues in children with ADHD has a considerable detrimental impact on their developmental trajectory. However, attention also represents an aspect that can be effectively improved through intervention, thereby exerting a positive influence on academic performance (Stevens and Bavelier, 2012). Consequently, it is important to intervene and ameliorate attention problems in school-age children.

Due to the ability of the stimulant medication to reduce ADHD symptoms in the short term, medication is currently the most common treatment for ADHD in children, often being chosen as a first-line treatment (Carucci et al., 2021; Mechler et al., 2022; Quenneville et al., 2022). However, medication treatment may be accompanied by side effects such as headaches, stomach pain, and decreased appetite (Pang and Sareen, 2021). Because of the potential side effects associated with medication treatment, non-pharmacological treatments with relatively lower side effects have developed rapidly in recent years (Cortese et al., 2022), such as physical activity interventions (Kleeren et al., 2023), neurofeedback interventions (Garcia Pimenta et al., 2021), and cognitive interventions (Pauli-Pott et al., 2021). Among them, physical activity interventions have become widely used non-pharmacological interventions due to their low cost, ease of operation, improvement of physical fitness, and long-lasting benefits including the acquisition of sports skills such as baseball techniques, equestrianism, basketball techniques, improvements in physical qualities such as strength, speed, flexibility, as well as a reduction in psychological issues and behavioral problems such as depression, anxiety, hyperactivity, and impulsivity (Kandola et al., 2019; Li et al., 2023a; Kleeren et al., 2023).

Physical activity is any physiological action that requires energy expenditure and is produced by the skeletal muscles (Bull et al., 2020). Additionally, physical activity requires a certain level of cognitive focus, and children with ADHD may struggle with maintaining attention, making it challenging for them to engage in sustained physical activities. Additionally, ADHD often co-occurs with other psychiatric conditions such as tic disorders (Banaschewski et al., 2007), which can contribute to motor impairments (Allen et al., 2005). Moreover, previous research has indicated that individuals with ADHD may experience a lack of physical activity due to a lack of patience and persistence in engaging in exercise (Chan et al., 2022). Therefore, physical activity interventions for individuals with ADHD have a dual significance in promoting both their physical and mental wellbeing (Harvey et al., 2009). Previous studies have considered ADHD to be caused by catecholamine dysfunction, and physical activity can affect the same dopaminergic and noradrenergic systems, thus producing corresponding physiological changes (Christiansen et al., 2019). Multiple studies have shown that physical activity interventions are a viable behavioural treatment option for ADHD children and adolescents, with good therapeutic effects in multiple aspects such as cognition, motor performance, and social problems (Ash et al., 2017; Den Heijer et al., 2017; Neudecker et al., 2019; Li et al., 2023a). Some previous meta-analyses have also demonstrated the beneficial therapeutic effects of physical activity on ADHD patients. In a study conducted by Zang et al. (2019), the effects of physical activity interventions *versus* nonphysical activity interventions in children with ADHD were assessed. The findings revealed that physical activity interventions had a significant positive impact on anxiety and depression, aggressive behaviour, thinking, and social problems among children with ADHD (Zang, 2019). Meanwhile, Lambez et al. (2019) conducted a meta-analysis to evaluate the effects of nonpharmacological treatments for ADHD on cognitive functioning, including neurofeedback, cognitive behavioural therapy, cognitive training, and physical exercise. The study found that physical exercise had the largest mean effect size, particularly for inhibition (Lambez et al., 2020). Welsch et al. (2020) conducted a meta-analysis on several aspects, including attention and working memory, in children with ADHD. They found that physical activity interventions had positive effects on attention, working memory, and other related aspects. Furthermore, they observed that physical activities with higher cognitive demands showed relatively greater benefits. Xiao et al. (2021) conducted a systematic review and meta-analysis to investigate the impact of exercise interventions on executive function in children and adolescents with ADHD. They found that moderate-intensity chronic exercise can improve executive function, but the difference in effects between children and adolescents could not be determined (Liang et al., 2021). Chueh et al. (2022) conducted a meta-analysis to assess the impact of acute MVPA on executive function in children with ADHD. Their findings suggest that acute MVPA can be an effective non-pharmacological treatment for improving ADHD symptoms (Chueh et al., 2022). Collectively, these findings suggest that physical exercise may represent an effective treatment strategy for individuals diagnosed with ADHD.

The current meta-analyses have mainly pooled children and adolescents together, without considering potential age-related differences, and have not specifically explored attention problems. However, given the rapid growth and development of children and adolescents and their significant physiological differences (Janssen and LeBlanc, 2010), attention problems exhibited by children with ADHD require further investigation into suitable types of physical activity interventions, intervention time, frequency, and period to develop more targeted intervention plans for precise treatment. This study conducts a meta-analysis of RCTs investigating physical activity interventions for attention problems in school-age children with ADHD, providing some insights for selecting effective physical activity intervention strategies to improve attention problems in this population.

## 2 Methods

### 2.1 Protocol and registration

We conducted this meta-analysis by the guidelines outlined in the Cochrane Handbook for Systematic Reviews of Interventions, and the results were reported using the Preferred Reporting Items for Systematic Reviews and Meta-Analyses (PRISMA) statement (Higgins et al., 2019; Page et al., 2021). Furthermore, we prospectively registered this meta-analysis in PROSPERO (CRD 42022363255).

### 2.2 Data sources and search strategy

We conducted a thorough search with no time limit across four databases, including PubMed, Web of Science, Embase, and the Cochrane Library, to identify relevant studies on physical activity interventions, age ranges, and outcomes related to patients with ADHD. The search terms for each main concept were developed based on previous reviews and expert opinions in the field of physical activity interventions (Tan et al., 2016; De Greeff et al., 2018; Xue et al., 2019). The search was performed up to October 2022 and was re-searched in May 2023; however, no additional literature that satisfied the inclusion criteria was discovered. Partial retrieval keywords are as follows: “Child” or “Children” and “Exercise” or “Physical Activity” or “Physical Exercise” or “Acute Exercise” or “Isometric Exercise” or “Aerobic Exercise” or “Exercise Training” and “ADHD” or “Attention Deficit Disorder with Hyperactivity” or “Attention Deficit Disorders” or “Attention Deficit Hyperactivity Disorder”. The detailed search strategy for the database can be found in Table 1 and Supplementary Appendix SA.

**TABLE 1:** Search strategy on PubMed.

#1	“ Attention deficit disorder with hyperactivity” [MeSH]
#2	(((((((((((((((((((((((Attention Deficit Disorder with Hyperactivity [Title/Abstract]) OR (Attention Deficit Disorders with Hyperactivity [Title/Abstract])) OR (ADHD [Title/Abstract])) OR (Attention Deficit Hyperactivity Disorder [Title/Abstract])) OR (Hyperkinetic Syndrome [Title/Abstract])) OR (Syndromes, Hyperkinetic [Title/Abstract])) OR (Attention Deficit-Hyperactivity Disorder [Title/Abstract])) OR (Attention Deficit-Hyperactivity Disorders [Title/Abstract])) OR (Deficit-Hyperactivity Disorder, Attention [Title/Abstract])) OR (Deficit-Hyperactivity Disorders, Attention [Title/Abstract])) OR (Disorder, Attention Deficit-Hyperactivity [Title/Abstract])) OR (Disorders, Attention Deficit-Hyperactivity [Title/Abstract])) OR (ADDH [Title/Abstract])) OR (Attention Deficit Hyperactivity Disorders [Title/Abstract])) OR (Attention Deficit Disorder [Title/Abstract])) OR (Attention Deficit Disorders [Title/Abstract])) OR (Deficit Disorder, Attention [Title/Abstract])) OR (Deficit Disorders, Attention [Title/Abstract])) OR (Disorder, Attention Deficit [Title/Abstract])) OR (Disorders, Attention Deficit [Title/Abstract])) OR (Brain Dysfunction, Minimal [Title/Abstract])) OR (Dysfunction, Minimal Brain [Title/Abstract])) OR (Minimal Brain Dysfunction [Title/Abstract]))
#3	#1 OR #2
#4	“ Child " [MeSH]
#5	((Child [Title/Abstract]) OR (Children [Title/Abstract]))
#6	#4 OR #5
#7	“ Exercise " [MeSH]
#8	((((((((((((((((((((((((((Exercise [Title/Abstract]) OR (Exercises [Title/Abstract])) OR (Physical Activity [Title/Abstract])) OR (Activities, Physical [Title/Abstract])) OR (Activity, Physical [Title/Abstract])) OR (Physical Activities [Title/Abstract])) OR (Exercise, Physical [Title/Abstract])) OR (Exercises, Physical [Title/Abstract])) OR (Physical Exercise [Title/Abstract])) OR (Physical Exercises [Title/Abstract])) OR (Acute Exercise [Title/Abstract])) OR (Acute Exercises [Title/Abstract])) OR (Exercise, Acute [Title/Abstract])) OR (Exercises, Acute [Title/Abstract])) OR (Exercise, Isometric [Title/Abstract])) OR (Exercises, Isometric [Title/Abstract])) OR (Isometric Exercises [Title/Abstract])) OR (Isometric Exercise [Title/Abstract])) OR (Exercise, Aerobic [Title/Abstract])) OR (Aerobic Exercise [Title/Abstract])) OR (Aerobic Exercises [Title/Abstract])) OR (Exercises, Aerobic [Title/Abstract])) OR (Exercise Training [Title/Abstract])) OR (Exercise Trainings [Title/Abstract])) OR (Training, Exercise [Title/Abstract])) OR (Trainings, Exercise [Title/Abstract]))
#9	#7 OR #8
#10	#3 AND #6 AND #9

### 2.3 Study selection

Two authors (DL and DW) independently assessed the search results and screened the publications retrieved from the databases, and also manually searched the reference lists of the included studies. The titles and abstracts of the studies were first screened to determine their relevance. Then, relevant studies underwent a full-text review to determine inclusion. Any discrepancies or inconsistencies were resolved through group discussion.

### 2.4 Inclusion and exclusion criteria

This systematic review employed specified inclusion criteria. The studies meeting the following criteria will be included:

(1) Only RCTs were included. RCTs were given preference due to their higher level of evidence-based ranking in the hierarchy of evidence (Brighton et al., 2003). Additionally, as meta-analysis involves a quantitative analysis of integrated literature, the quality of the included studies influences the credibility of the Meta-analysis. By selecting RCTs with a control group and randomization, we aimed to minimize bias and effectively address the research question concerning the effectiveness of physical activity interventions on the population. Thus, the decision to exclusively include RCTs was made.
(2) The range of age participants in the sample must be 6–12 years. The school-age period is the stage with the highest rates of ADHD diagnosis (First, 2013) and is also a crucial stage in the academic development. The behavioral issues associated with ADHD persist into adolescence and adulthood, thereby impacting long-term outcomes (American Psychiatric Association and American Psychiatric, 2013). Consequently, early intervention targeting school-age children with ADHD holds significant importance for individuals within the ADHD population.
(3) The physical activity intervention had to contain a sports or physical activity component.
(4) The study must report data on indicators of attention problems in children with ADHD before and after the intervention.
(5) Presented original data.
(6) We only analysed papers written in English and excluded papers written in other languages.

Studies were excluded if they:

(1) were based on observational studies, including cross-sectional, case-control, and cohort designs.
(2) The age range of the study participants was less than 6 years and greater than 12 years.
(3) Studies without physical activity intervention.
(4) Studies that do not report attention problems.
(5) No raw data were provided.
(6) Publications written in a language other than English were excluded.

### 2.5 Data extraction

The extracted data was organized in a standardized Excel spreadsheet. The data from the included trials were extracted by two authors (DL and DW) independently, and any discrepancies arising during the process were resolved through group discussion. The following information was extracted from each study: author, year, country, participant characteristics, intervention characteristics, and relevant indicators of attention problems in school-age children with ADHD. When encountering unclear post-intervention results but presented in graphical form, we utilized the Engauge Digitizer software to extract the data. For studies with multiple follow-up assessments, we only extracted data immediately after the intervention. In cases where the Standard Deviation was not provided, we calculated the standard deviation using the confidence interval (95%) of the mean within either the intervention or control group.

Based on the included studies, we classified interventions into two categories based on previous research. Activities that require high cognitive engagement and occupy more than 50% of total physical activity time, such as soccer, shooting, and water sports, were classified as CEE (cognitively engaging exercise), while regular running and jumping exercises were classified as AE (aerobic exercise) (Welsch et al., 2021; Sung et al., 2022). We further categorized interventions based on the interference environment in which they took place. For example, the instability of the water environment in water sports and the random variability on the field in soccer were classified as EFY (environmentally fluctuating type), while treadmill running and yoga practice were classified as EST (environmentally stable type). With this classification, we aim to investigate whether the attention of school-age children with ADHD is influenced differently by the interference environment of the intervention content. The type of control group was classified as OI (other intervention) or NI (no intervention). The intervention time was categorized as “60 min and above” and “Less than 60 min”. The intervention frequency was categorized as “3 times per week and above” and “Less than 3 times per week”. The intervention period was categorized as “More than 12 weeks”, “Between 9 and 12 weeks”, and “Less than or equal to 8 weeks”.

### 2.6 Quality assessment

The risk of bias was evaluated using the Cochrane Risk of Bias Assessment Tool within the Review Manager 5.4 software, which evaluates the studies’ quality on seven indicators: 1. Random sequence generation; 2. Allocation concealment; 3. Blinding of participants and personnel; 4. Blinding of outcome assessment; 5. Incomplete outcome data; 6. Selective reporting; and 7. Other bias.

### 2.7 Certainty assessment

The credibility of the results is assessed using the GRADE (Grading of Recommendations, Assessment, Development, and Evaluation) framework, which provides recommendations for evaluation, development, and assessment. The quality of evidence is examined in the following domains: Risk of bias, inconsistency, indirectness, and imprecision (Balshem et al., 2011).

### 2.8 Statistical analysis

As the included data were continuous outcomes, we calculated the standardized mean difference (SMD) and 95% confidence interval (CI). Effect sizes were interpreted according to convention, where an SMD of ≤0.2 represents a small effect, ≤0.5 represents a medium effect, and ≤0.8 represents a large effect (Khera et al., 2016). We used the Cochrane Q test to assess the degree of statistical heterogeneity across trials, which was quantified using the *I* ^*2*^ statistic. We categorized the level of heterogeneity as low (*I* ^*2*^ ≤ 25%), moderate (25% < *I* ^*2*^ ≤ 50%), substantial (50% < *I* ^*2*^ ≤ 75%), or considerable (*I* ^*2*^ > 75%) (Higgins et al., 2003). We selected different effect models based on the level of heterogeneity in the trial data. When the level of heterogeneity was low, we used a fixed-effects model (*p* ≥ 0.1 and *I* ^*2*^ ≤ 50%) to analyze the data; otherwise, we used a random-effects model (*p* < 0.1 or *I* ^*2*^ > 50%) (Khera et al., 2016).

We conducted subgroup analyses based on categorical variables, including different types of physical activity, intervention duration, intervention frequency, and intervention period, to investigate potential sources of heterogeneity. We also created funnel plots of the outcome indicators and conducted symmetry tests to investigate potential publication bias and the impact of small sample studies on outcome indicators. Additionally, we performed sensitivity analyses by progressively removing individual studies to assess the robustness of our research findings (Khera et al., 2016). We performed all statistical analyses using Stata15.1 with two-sided tests and considered *p* values < 0.05 to be statistically significant.

## 3 Results

### 3.1 Trial selection

To ensure the accuracy of the systematic retrieval process, two reviewers (DL and DW), who are familiar with ADHD attention issues and research in the field of sports science, conducted the retrieval process and independently screened titles, abstracts, and full-text articles. The inter-rater reliability (Cohen’s kappa) was calculated for both stages of review, including the screening of titles and abstracts, as well as the full-text screening. The level of agreement was classified as follows: fair agreement [0.40–0.59], good agreement [0.60–0.74], and excellent agreement [> 0.75] (Sim and Wright, 2005; Vickers, 2017).

**FIGURE 1:**
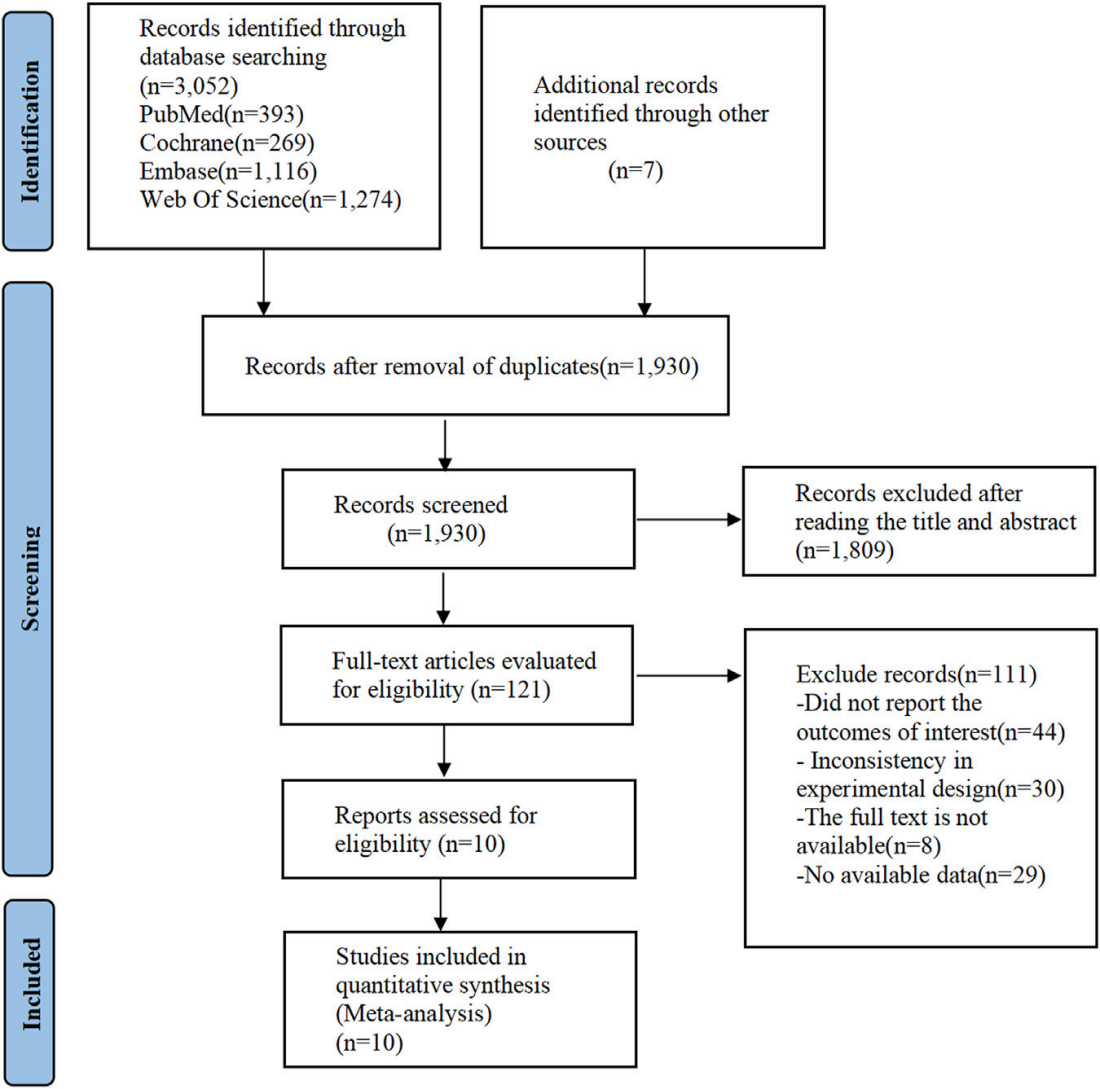
PRISMA flow diagram of the study process.

In the initial search of electronic databases, a total of 3,052 citations were obtained, and an additional 7 documents were manually searched. After removing duplicate studies (n = 1,129), 1930 relevant articles remained. Subsequently, 1809 articles were excluded through screening, leaving 121 articles suitable for full-text review. The inter-rater reliability between the two reviewers at this stage was classified as good (Cohen’s kappa = 0.68). After full-text reading, 111 articles were further excluded, of which 44 did not report the outcomes of interest, 30 had inconsistency in experimental design, 8 had no full-text available, and 29 had no available data. Finally, 10 studies (Jensen and Kenny, 2004; Kang et al., 2011; Hoza et al., 2015; Bustamante et al., 2016; García-Gómez et al., 2016; Pan et al., 2016; Geladé et al., 2017; Oh et al., 2018; Silva et al., 2020; Ahn et al., 2021) were included for quantitative synthesis (Figure 1). The inter-rater reliability between the two reviewers at this stage was classified as good (Cohen’s kappa = 0.79).

### 3.2 Trial characteristics

Table 2 presents the characteristics of the included studies, which comprised a total of 10 studies. All studies were published between 2004 and 2021 and utilized a randomized controlled trial design. The country with the highest number of included studies was South Korea, with a total of 3 papers. The sample sizes for the intervention groups ranged from 8 to 104 individuals, totalling 245 school-aged children with ADHD. The sample sizes for the control groups ranged from 5 to 98 individuals, totalling 229 school-aged children with ADHD. The age range for both the intervention and control groups was 6–12 years old, with a higher proportion of boys included in the studies. All studies utilized the Diagnostic and Statistical Manual of Mental Disorders (DSM) (4th or 5th edition) for standardized diagnosis.

**TABLE 2:** Summary table of included reviews.

Study	Country	Sample size		Gender (M/F)	Mean Age (year)		Intervention						Outcome diagnostics	ADHD diagnostics
		EG	CG		EG	CG	EG			CG				
							Intervention content	Intervention time, frequency, period (intensity)	Type	Intervention content	Intervention time, frequency, period	Type		
Oh et al. (2018)	Korea	17	17	31/3	8.30	8.00	Horsemanship Practice	40 min, 2 weekly, 12 weeks (NR)	CEE	Pharmacotherapy	Consistent with EG	PCT	Korean version of the ADHD Rating Scale	DSM-4
Ahn et al. (2021)	Korea	8	7	12/3	7.50	7.14	Horsemanship Practice	40 min, 2 weekly, 16 weeks (NR)	CEE	NI	NI	NI	DuPaul’s ADHD Rating Scale	DSM-5
Silva et al. (2019)	Brazil	10	10	14/6	12	12.00	Swimming–learning program	45 min, 2 weekly, 8 weeks (NR)	CEE	NI	NI	NI	Cancellation Attention Test	DSM-4
Kang et al. (2011)	Korea	15	13	28/0	8.4	8.6	Sports therapy (aerobic exercise and goal-directed exercise)	90 min, 2 weekly, 6 weeks (NR)	CEE	Education for behavior	NR	CI	DuPaul’s ADHD Rating Scale	DSM-4
Bustamante et al. (2016)	America	18	16	24/10	9.40	8.70	Physically active games (physically active games)	90 min, 5 weekly, 10 weeks (75% of HRmax)	AE	Sedentary task	Consistent with EG	CI	Impairment Rating Scale	DSM-4
Pan et al. (2016)	China	16	16	32/0	8.93	8.87	Table tennis exercise	70 min, 2 weekly, 12 weeks (NR)	CEE	NI	NI	NI	Chinese version of the Child Behavior Checklist	DSM-4
Geladé et al. (2016)	Netherlands	37	39	58/18	9.80	9.96	Aerobic exercise (warming up + exercises)	20 min, 3 weekly, 10–12 weeks	AE	Neurofeedback (Theta/beta training)	Consistent with EG	NFB	Auditory oddball task	DSM-4
Garcia-Gómez et al. (2016)	Spain	9	5	10/4	10.65	10.20	Equestrian therapy	45 min, 2 weekly, 12 weeks, (NR)	CEE	NI	NI	NI	Behavior Assessment System for Children	DSM-4
Hoza et al. (2015)	America	104	98	108/94	6.83	6.83	Aerobic exercise (Structured fun aerobic activities)	31 min, 5 weekly, 12 weeks (moderate-to-vigorous range)	AE	sedentary task	Consistent with EG	CI	Conners Parent and Teacher Rating Scale	DSM-4
Jensen and Kenny et al. (2004)	Australia	11	8	19/0	10.63	9.35	Yoga	60 min, 1 weekly, 20 weeks (NR)	CEE	Cooperative games	Consistent with EG	CI	Conners Parent and Teacher Rating Scale	DSM-4

ADHD, attention-deficit/hyperactivity disorder; EG, experimental group; CG, control group; NR, no report; NI, no intervention; HR, heart rate; NFB, neurofeedback; CI, cognitive intervention; PCT, pharmacotherapy; DSM-4 and DSM-5, diagnostic and statistical manual of mental disorders, fourth edition and fifth edition.

In general, the intervention strategies can be classified into two categories: CEE (6 studies) and AE (4 studies). The time of a single intervention ranged from 20 to 90 min, and the intervention frequency ranged from once a week to five times a week. The intervention period ranged from 6 to 20 weeks. Additionally, only two studies reported on the intensity of physical activity, both of which were classified as moderate-to-vigorous physical activity (MVPA). Attention was typically measured using tools such as the DuPaul’s ADHD Rating Scale, Conners Parent and Teacher Rating Scale, Behavior Assessment System for Children, Auditory oddball task, and Impairment Rating Scale.

### 3.3 Risk of bias

**FIGURE 2:**
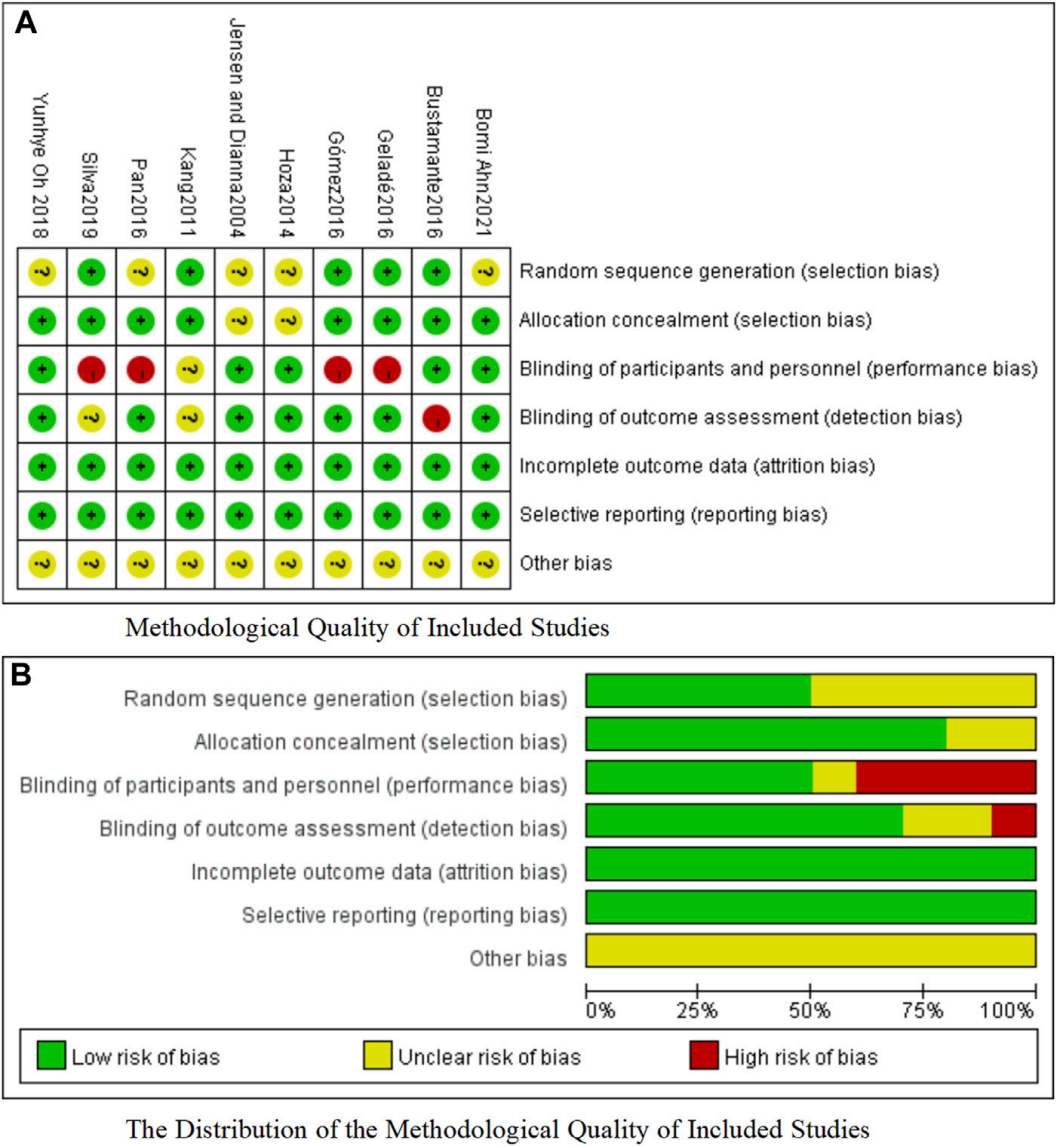
**(A)** Methodological quality of included studies. **(B)** The distribution of the methodological quality of included studies.

Five studies (Kang et al., 2011; Bustamante et al., 2016; García-Gómez et al., 2016; Geladé et al., 2017; Silva et al., 2020) (50.0%) had a low risk of bias with respect to random sequence generation. Eight studies (Kang et al., 2011; Bustamante et al., 2016; García-Gómez et al., 2016; Pan et al., 2016; Geladé et al., 2017; Oh et al., 2018; Silva et al., 2020; Ahn et al., 2021) (80.0%) had a low risk of bias with respect to allocation concealment. Five studies (Jensen and Kenny, 2004; Hoza et al., 2015; Bustamante et al., 2016; Oh et al., 2018; Ahn et al., 2021) (50.0%) had a low risk of bias with respect to the blinding of participants and personnel. Seven studies (Jensen and Kenny, 2004; Hoza et al., 2015; García-Gómez et al., 2016; Pan et al., 2016; Geladé et al., 2017; Oh et al., 2018; Ahn et al., 2021) (70.0%) had a low risk of bias with respect to the blinding of outcome assessments. Ten studies (Jensen and Kenny, 2004; Kang et al., 2011; Hoza et al., 2015; Bustamante et al., 2016; García-Gómez et al., 2016; Pan et al., 2016; Geladé et al., 2017; Oh et al., 2018; Silva et al., 2020; Ahn et al., 2021) (100.0%) had a low risk of bias with respect to incomplete outcome data. Ten studies (Jensen and Kenny, 2004; Kang et al., 2011; Hoza et al., 2015; Bustamante et al., 2016; García-Gómez et al., 2016; Pan et al., 2016; Geladé et al., 2017; Oh et al., 2018; Silva et al., 2020; Ahn et al., 2021) (100.0%) had a low risk of bias with respect to selective reporting. Other biases are not known. Details of the evaluation of bias results for the included literature are shown in Figures 2A, B.

### 3.4 Certainty assessment results

According to the GRADE approach, the assessment results for each domain are as follows: the risk of bias, indirectness, and imprecision domains are not considered serious, while the inconsistency domain is considered serious. The detailed GRADE approach is presented in Table 3.

**TABLE 3:** GRADE.

Certainty assessment						No of patients		Effect	Importance
No of studies	Study design	Risk of bias	Inconsistency	Indirectness	Imprecision	[EG]	[CG]	Absolute (95% CI)	
10	randomised trials	not serious	serious	not serious	not serious	245	229	SMD -0.46 (−0.85 to −0.07)	CRITICAL

EG, experimental group; CG, control group; CI, confidence interval; SMD, standardised mean difference.

### 3.5 Meta-analysis

#### 3.5.1 Meta-analysis of effects of physical activity on attention problems

**FIGURE 3:**
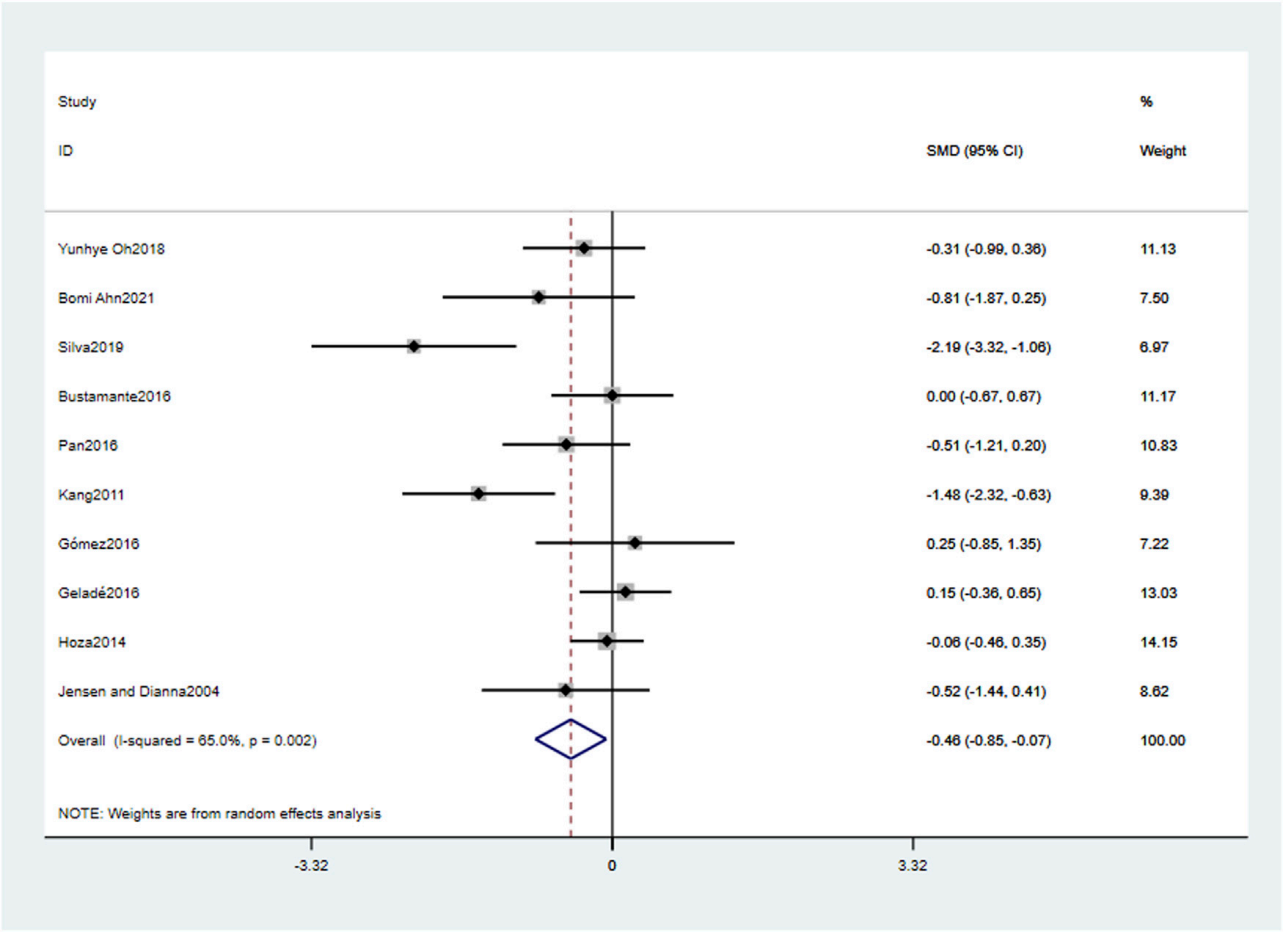
Forest plot for meta-analysis regarding the effect of physical activity interventions on attention problems.

A total of ten studies reported the effect of physical activity on attention problems. As shown in Figure 3, the overall effect indicates a statistically moderate effect size (SMD = −0.48, 95% CI: 0.85, −0.07, *p* < 0.05) of physical activity interventions in improving attention problems in children with ADHD compared to the control group, with large heterogeneity (*I* ^*2*^ = 65.0%, *p* < 0.05). This suggests that physical activity interventions have a certain degree of improvement effect on attention problems in children with ADHD.

#### 3.5.2 Moderator analysis

As shown in the analysis results of Figure 3, physical activity has a significant overall moderating-moderate effect on school-age children with ADHD (SMD = −0.48, 95% CI: 0.85, −0.07, *p* < 0.05), with large heterogeneity (*I* ^*2*^ = 65.0%, *p* < 0.05). Thus, the high heterogeneity indicates that we can interpret its variability through moderator analysis. To investigate potential moderating effects, we conducted a subgroup analysis based on intervention type, the content of control group intervention, physical activity environment, intervention time, intervention frequency, and intervention period.

**FIGURE 4:**
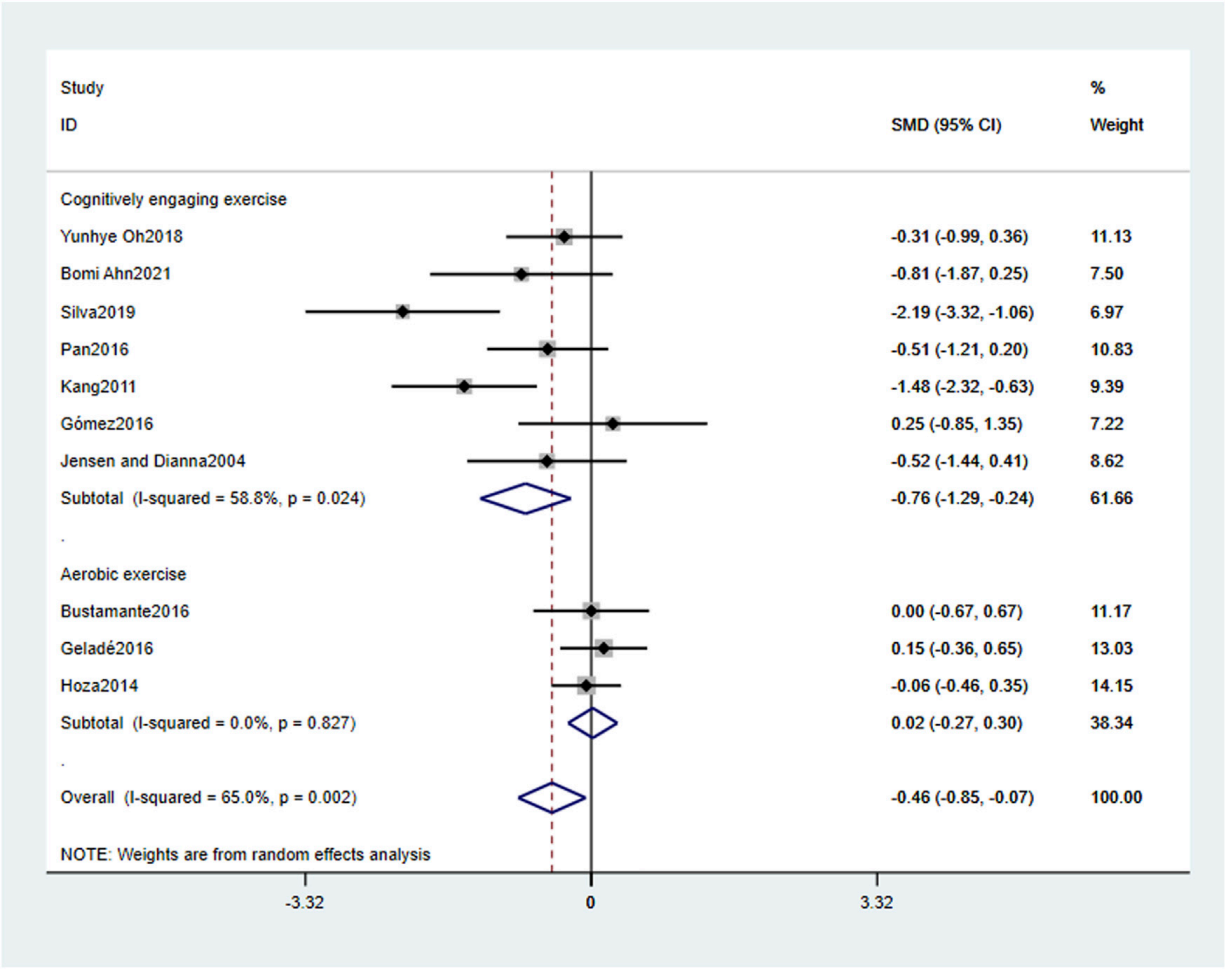
The subgroup analysis of the effect of physical activity intervention type on attention problems.

**FIGURE 5:**
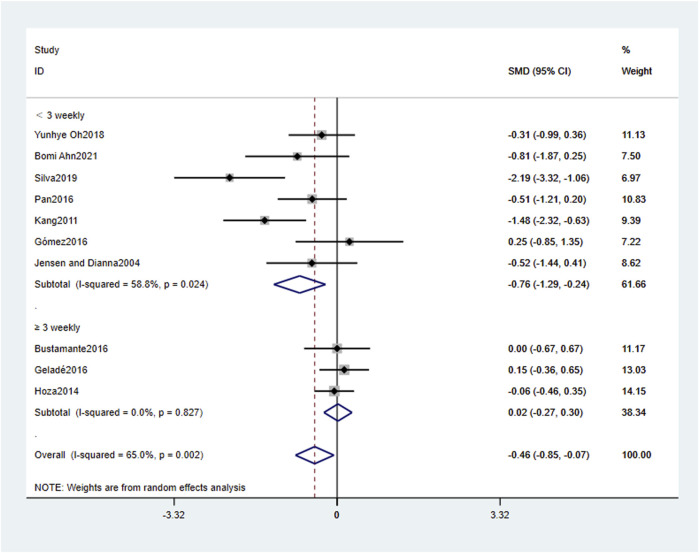
The subgroup analysis of the effect of physical activity intervention frequency on attention problems.

**FIGURE 6:**
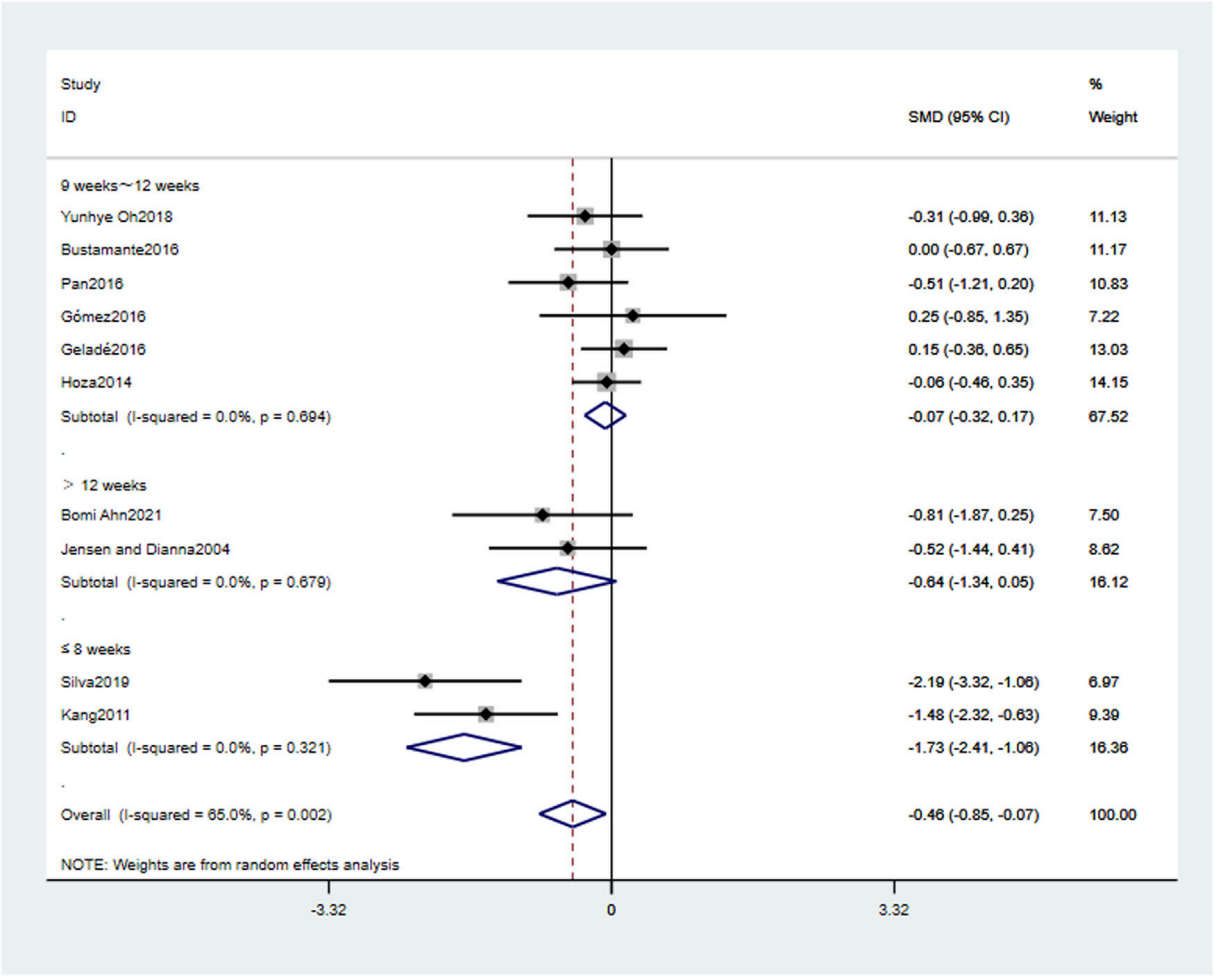
The subgroup analysis of the effect of physical activity intervention period on attention problems.

Table 4 and Figures 4–6 present the results of the subgroup analysis regarding attention problems. The results show that the effect of physical activity intervention is moderated by intervention type, intervention frequency, and intervention period, but not affected by physical activity environment or single intervention time. The type of physical activity intervention “cognitively engaging exercise (CEE)" had a large effect size (SMD = −0.76, 95% CI: 0.76, −0.24, *p* < 0.01), with large heterogeneity (*I* ^*2*^ = 58.8%, *p* < 0.05). The frequency of physical activity intervention “Less than 3 weekly” had a large effect size (SMD = −0.76, 95% CI: 1.29, −0.24, *p* < 0.01), with large heterogeneity (*I* ^*2*^ = 58.8%, *p* < 0.05). The period of physical activity intervention “Less than or equal to 8 weeks” had a large effect size (SMD = −1.73, 95% CI: 2.41, −1.06, *p* < 0.01), with small heterogeneity (*I* ^*2*^ = 0.0%, *p* > 0.05).

**TABLE 4:** The subgroup analysis of the effect of physical activity interventions on attention problems.

Subgroup	Cut-off	Inclusion of literature	Heterogeneity test		SMD (95% CI)	P
			I^2^%	P		
Type	CEE	7	58.8	0.024	−0.76 (-0.76, −0.24)	0.004**
	AE	3	0.0	0.827	0.02 (-0.27,0.30)	0.906
Environment	EFY	4	72.1	0.013	−0.73 (-1.65,0.19)	0.119
	EST	6	60.2	0.028	−0.32 (-0.73,0.09)	0.131
Control group	OI	6	58.0	0.036	−0.28 (-0.68,0.12)	0.165
	NI	4	69.6	0.020	−0.79 (-1.68,0.10)	0.083
Time	≥60 min	4	58.4	0.066	−0.59 (-1.20,0.01)	0.054
	<60 min	6	69.1	0.006	−0.38 (-0.90,0.15)	0.163
Frequency	≥3 weekly	3	0.0	0.827	0.02 (-0.27,0.30)	0.906
	<3 weekly	7	58.8	0.024	−0.76 (-1.29, −0.24)	0.004**
Period	>12 weeks	2	0.0	0.679	−0.64 (−1.34,0.05)	0.071
	9–12 weeks	6	0.0	0.694	−0.07 (-0.32, 0.17)	0.544
	≤8 weeks	2	0.0	0.321	−1.73 (-2.41, −1.06)	0.001**

*: *p* ≤ 0.05; **: *p* ≤ 0.01; CEE, cognitively engaging exercise; AE, aerobic exercise; EFY, environmental fluctuation type; EST, environmentally stable type; NI, no intervention; OI, other intervention.

### 3.6 Publication bias

As demonstrated in Supplementary Appendix SB1, the use of funnel plots enabled us to detect any potential publication bias. Upon visual inspection of the plots for attention problems, no significant evidence of publication bias was detected. In Supplementary Appendix SB2, a sensitivity analysis conducted through iterative removal of individual studies did not reveal any influential studies that significantly impacted the overall results, indicating the robustness of our findings.

## 4 Discussion

We conducted a systematic review and meta-analysis of 10 RCTs to examine the effects of physical activity interventions on attention problems in school-age children with ADHD. Our study found that physical activity has an overall positive effect on attention problems in school-age children with ADHD, with cognitively engaging exercise being more helpful for improving attention problems than traditional aerobic exercise. The type of physical activity intervention, including cognitively engaging exercise, intervention frequency less than 3 times per week, and intervention period of less than 8 weeks, showed statistically significant effects. This indicates that physical activity interventions in this form can achieve the best intervention effect and may be the most favourable physical activity design for improving attention problems in school-age children with ADHD.

As for the effects of physical activity interventions, our study is consistent with some previous research findings. Physical activity can improve participants’ attention problems, social abilities, and executive functions (Verburgh et al., 2014; Ludyga et al., 2016; De Greeff et al., 2018), and can produce numerous beneficial effects on physiological, psychological, and neurocognitive aspects, including reductions in stress, anxiety, depression, and negative emotions (Tsatsoulis and Fountoulakis, 2006; McKercher et al., 2009; Garcia et al., 2012; Diamond, 2015; Li et al., 2023b). Physical activity interventions also have a certain degree of improvement effect on the symptoms of ADHD patients, previous studies have shown that ADHD patients have multiple core symptoms improved after participating in long-term physical activity, including decreased attention problems, improved cognitive functions, increased coordination, and reduced behavioural problems (Niederer et al., 2011; Verret et al., 2012; Choi et al., 2015; Pan et al., 2016; Pan et al., 2019). Jensen et al. (2004) conducted a yoga intervention for boys with ADHD and found significant improvements in attention, while overactivity, anxiety/shyness, and social problems did not show significant improvement (Jensen and Kenny, 2004). Implemented a table tennis exercise intervention for boys with ADHD and observed positive improvements in attention and executive functions (Pan et al., 2016). Therefore, multiple studies indicate that physical activity has a beneficial impact on attention issues in school-age children with ADHD. However, further research is needed to explore the potential benefits in other aspects for this population.

Regarding the mechanisms by which physical activity improves attention, there is evidence to suggest that physical activity can act as a neuroenhancer, increasing cognitive abilities through both acute and long-term effects on monoaminergic transmission, neural nutrition signalling, and neural plasticity mechanisms (Hillman et al., 2008). Additionally, participating in physical activity can improve brain function, such as memory and attention, in children due to factors released by contracting muscles, such as metabolites and myokines (Van Praag, 2009; Di Liegro et al., 2019). This has led to some degree of improvement in attention issues in children with ADHD who engage in physical activity.

In our study, we found that cognitively engaging exercise is more effective than traditional aerobic exercise in improving attentional problems in school-age children with ADHD. Cognitively engaging exercise involves physical activities with high levels of cognitive engagement, requiring greater cognitive control and more varied environmental stimuli, such as ball games, horseback riding, and water sports (Tomporowski et al., 2015). The mechanism underlying the positive effect of cognitively engaging exercise on attentional problems in ADHD children may be related to its characteristics. Compared to traditional aerobic exercise, cognitively engaging exercise demands higher levels of cognitive control and greater environmental variability, necessitating additional attention to rapidly changing situations and requiring participants to perform tasks in various environments (Hung et al., 2018; Liang et al., 2021). These factors may contribute to the improvement of attentional problems and environmental adaptation in children with ADHD.

In our study, we found that the benefits of improved attention in school-age children with ADHD are not necessarily positively correlated with higher frequency and longer duration of physical activity. Both physical activity interventions and medication treatments may share a similarity in that higher dosage does not necessarily equate to better outcomes. The positive effects of physical activity interventions may decrease when administered beyond the optimal therapeutic range. Therefore, it is crucial to consider the importance of precision intervention by taking into account different subtypes, gender differences, and comorbidities within the ADHD population. Tailoring the type, frequency, duration, and duration of physical activity interventions according to individual characteristics will lead to the optimal treatment approach.

According to the aforementioned, physical activity has a significant effect on improving attention problems in school-aged children with ADHD. However, the effectiveness varies depending on the type, intervention time, frequency, and period of physical activity. Overall, physical activity is an effective non-pharmacological intervention for improving attention problems in school-aged children with ADHD. In particular, cognitively engaging exercise have better results in improving attention problems in this population.

## 5 Strengths and limitations

Our systematic review and meta-analysis on the effects of physical activity on attention problems in school-age children with ADHD have several strengths. First, we only included randomized controlled trials, which improves the reliability of our findings by excluding observational and cross-sectional studies. Second, our focus on the school-age developmental stage allows for more targeted and precise interventions. Third, our study provides valuable reference information for selecting physical activity therapies for attention problems in school-age children with ADHD.

However, our meta-analysis has some limitations that may affect the interpretation of our results. Firstly, the number of eligible studies was relatively small, which limits the statistical power and strength of our conclusions. Secondly, the limited data available for subgroup analysis may also impact our results. Thirdly, the majority of the participants included in the analysis were boys, and we cannot assess potential gender differences. Fourthly, we believe that the effectiveness of physical activity interventions may vary among the three subtypes of ADHD. However, due to the lack of studies that specifically differentiate interventions for each subtype, we were unable to explore potential differences in subtypes of ADHD. We hope that future researchers will take this into consideration and provide more precise interventions for ADHD based on subtypes. Finally, caution should be exercised when interpreting our findings due to the limited number of studies included, and further research is necessary to provide more robust evidence.

## 6 Conclusion

Our study suggests that cognitively engaging exercise is more effective in improving attention problems in school-aged children with ADHD. Specifically, when cognitive-engaging exercise is used as the type of physical activity and the intervention frequency is less than 3 times per week, with an intervention period of less than 8 weeks, it is most beneficial for improving attention problems in school-aged children with ADHD. However, we should also consider individual differences in children with respect to their ADHD symptoms and accurately evaluate each child’s specific symptoms before intervention.

## Data Availability

The original contributions presented in the study are included in the article/Supplementary Material, further inquiries can be directed to the corresponding author.
