# Supplementary material for: Effect of physical activity on attention in school-age children with ADHD: a systematic review and meta-analysis of randomized controlled trials
Source: Front Physiol. 2023 Jul 27;14:1189443. doi: 10.3389/fphys.2023.1189443 (PMC10415683; doi:10.3389/fphys.2023.1189443)
Supplement: Supplementary file 2 [file DataSheet2.docx]

**Effect of physical activity on attention in school-age children with ADHD: A systematic review and Meta-analysis of randomized controlled trials**

**Dong Li^1,2^**†**, Lan Li^3^**†**, Wanli Zang^4^, Deng Wang^5^, Chuyuan Miao^6^****, Chenmu Li^1^****^*^,** **Li Zhou****^7*^**

**Jin Yan^8,9^**

^1^School of Physical Education, Guangzhou Sport University, Guangzhou, China

^2^Department of International Culture Education, Chodang University, Republic of Korea

^3^Universuty of Maine at Presque Isle, Northern Maine, United States

^4^Postgraduate School, University of Harbin Sport, Harbin, China

^5^LFE Research Group, Department of Health and Human Performance. Universidad Politécnica de Madrid, Martín Fierro 7, Madrid, Spain

^6^School of Nursing, Guangzhou Medical University, Guangzhou, China

^7^School of Sports and Health, Guizhou Medical University, 550025, Guiyang, China

^8^Centre for Active Living and Learning, University of Newcastle, Callaghan, NSW, Australia

^9^College of Human and Social Futures, University of Newcastle, Callaghan, NSW, Australia

*** Correspondence:**Chenmu Li

[51229@gzsport.edu.cn](mailto:51229@gzsport.edu.cn)

Li Zhou

[zhouli@gmc.edu.cn](mailto:zhouli@gmc.edu.cn)

† These authors share the first authorship.

#### Appendix A

**A1:Web of science** **(n=1,274)**

**#1：(TS=(Child)) OR TS=(Children)**

**#2(((((((((((((((((((((((((TS=(Exercise)) OR TS=(Exercises)) OR TS=(Physical Activity)) OR TS=(Activities, Physical)) OR TS=(Activity, Physical)) OR TS=(Physical Activities)) OR TS=(Exercise, Physical)) OR TS=(Exercises, Physical)) OR TS=(Physical Exercise)) OR TS=(Physical Exercises)) OR TS=(Acute Exercise)) OR TS=(Acute Exercises)) OR TS=(Exercise, Acute)) OR TS=(Exercises, Acute)) OR TS=(Exercise, Isometric)) OR TS=(Exercises, Isometric)) OR TS=(Isometric Exercises)) OR TS=(Isometric Exercise)) OR TS=(Exercise, Aerobic)) OR TS=(Aerobic Exercise)) OR TS=(Aerobic Exercises)) OR TS=(Exercises, Aerobic)) OR TS=(Exercise Training)) OR TS=(Exercise Trainings)) OR TS=(Training, Exercise)) OR TS=(Trainings, Exercise)**

**#3(((((((((((((((((((TS=(Attention Deficit Disorder with Hyperactivity)) OR TS=(Attention Deficit Disorders with Hyperactivity)) OR TS=(ADHD)) OR TS=(Attention Deficit Hyperactivity Disorder)) OR TS=(Hyperkinetic Syndrome)) OR TS=(Syndromes, Hyperkinetic)) OR TS=(Attention Deficit-Hyperactivity Disorder)) OR TS=(Attention Deficit-Hyperactivity Disorders)) OR TS=(Deficit-Hyperactivity Disorder, Attention)) OR TS=(Deficit-Hyperactivity Disorders, Attention)) OR TS=(Disorder, Attention Deficit-Hyperactivity)) OR TS=(Disorders, Attention Deficit-Hyperactivity)) OR TS=(ADDH)) OR TS=(Attention Deficit Hyperactivity Disorders)) OR TS=(Attention Deficit Disorder)) OR TS=(Attention Deficit Disorders)) OR TS=(Deficit Disorder, Attention)) OR TS=(Deficit Disorders, Attention)) OR TS=(Disorder, Attention Deficit)) OR TS=(Disorders, Attention Deficit)**

**#1 and #2 and #3**

**A2:Cochrane** **(n=269)**

**
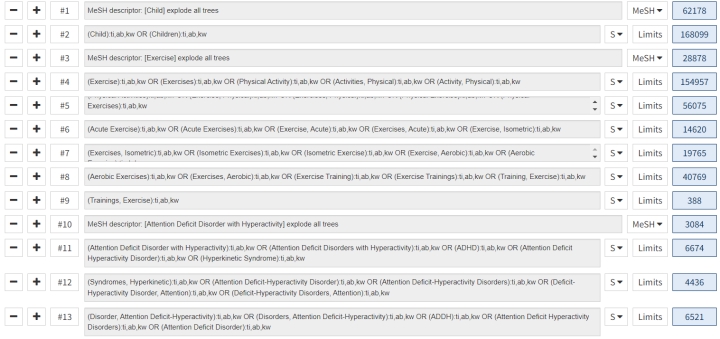
**

**
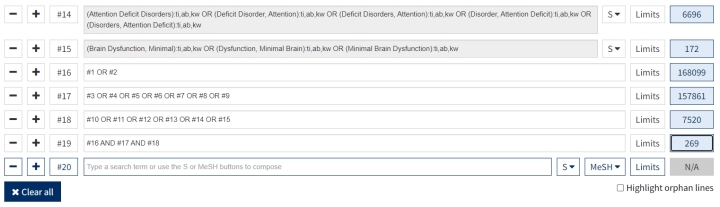
**

A3**:Embase (n=1,116)**

**
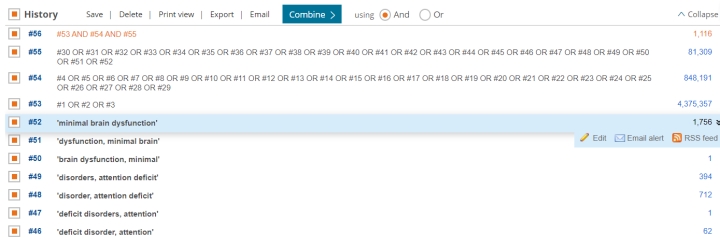
**

**
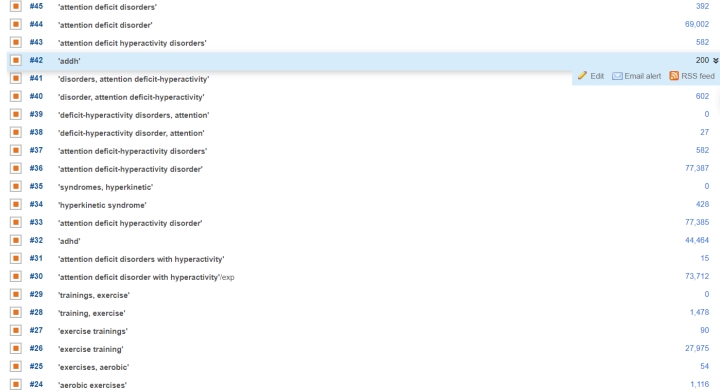
**

**
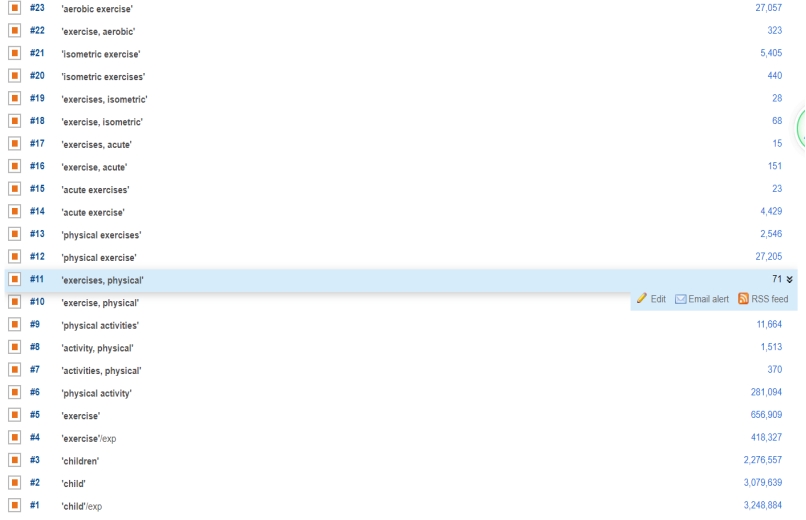
**

#### Appendix B


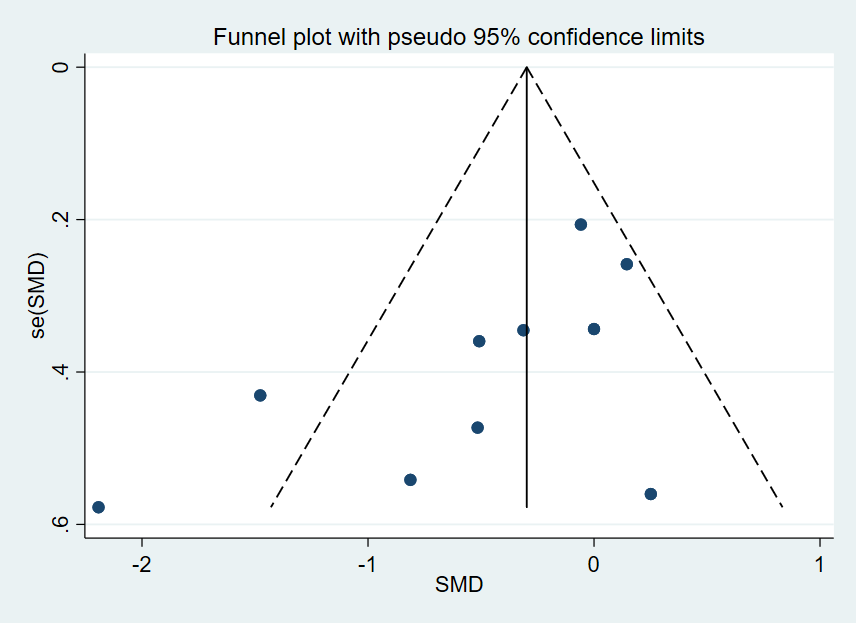


**B1** Funnel plot on publication bias.


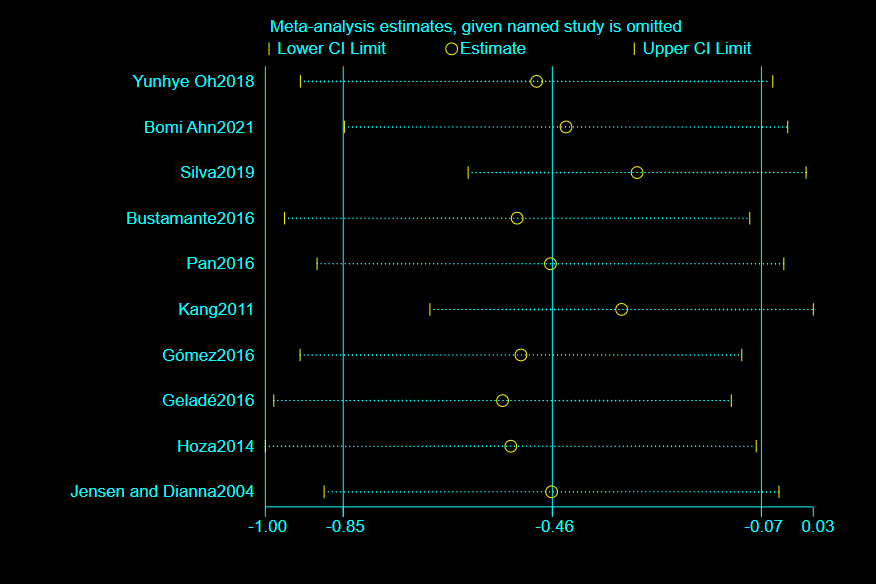


**B2 Sensitivity analysis.**
